# Immunization with DAT fragments is associated with long-term striatal impairment, hyperactivity and reduced cognitive flexibility in mice

**DOI:** 10.1186/1744-9081-8-54

**Published:** 2012-11-28

**Authors:** Walter Adriani, Susanne Koot, Sandra Columba-Cabezas, Emilia Romano, Domenica Travaglini, Ruud van den Bos, Oleg Granstrem, Syed F Ali, Giovanni Laviola

**Affiliations:** 1 Dept. Cell Biology & Neurosciences, Istituto Superiore di Sanità, Rome, Italy; 2 Dept. Neuroscience & Pharmacology, Rudolf Magnus Institute of Neuroscience, University Medical Center of Utrecht, Utrecht, the Netherlands; 3 Dept. Neurology & Neurosurgery, I.P. Pavlov’s State Medical University and Geropharm Ltd, St. Petersburg, Russia; 4 Neurochemistry Lab, Division of Neurotoxicology, National Center for Toxicological Research / FDA, Jefferson, AR, USA

**Keywords:** Auto-antibodies to neuro-receptors, DAT, Delay of reward, Flexibility of choice behaviour, ADHD, OCD

## Abstract

**Background:**

Possible interactions between nervous and immune systems in neuro-psychiatric disorders remain elusive. Levels of brain dopamine transporter (DAT) have been implicated in several impulse-control disorders, like attention deficit / hyperactivity disorder (ADHD) and obsessive-compulsive disorder (OCD). Here, we assessed the interplay between DAT auto-immunity and behavioural / neurochemical phenotype.

**Methods:**

Male CD-1 mice were immunized with DAT peptide fragments (DAT-i), or vehicle alone (VEH), to generate elevated circulating levels of DAT auto-antibodies (aAbs). Using an operant delay-of-reward task (20 min daily sessions; timeout 25 sec), mice had a choice between either an immediate small amount of food (SS), or a larger amount of food after a delay (LL), which increased progressively across sessions (from 0 to 150 sec).

**Results:**

DAT-i mice exhibited spontaneous hyperactivity (2 h-longer wake-up peak; a wake-up attempt during rest). Two sub-populations differing in behavioural flexibility were identified in the VEH control group: they showed either a clear-cut decision to select LL or clear-cut shifting towards SS, as expected. Compared to VEH controls, choice-behaviour profile of DAT-i mice was markedly disturbed, together with long-lasting alterations of the striatal monoamines. Enhanced levels of DA metabolite HVA in DAT-i mice came along with slower acquisition of basal preferences and with impaired shifting; elevation also in DOPAC levels was associated with incapacity to change a rigid selection strategy. This scarce flexibility of performance is indicative of a poor adaptation to task contingencies.

**Conclusions:**

Hyperactivity and reduced cognitive flexibility are patterns of behaviour consistent with enduring functional impairment of striatal regions. It is yet unclear how anti-DAT antibodies could enter or otherwise affect these brain areas, and which alterations in DAT activity exactly occurred after immunization. Present neuro-behavioural alterations, coming along with an experimentally-induced rise of circulating DAT-directed aAbs, open the issue of a potential role for auto-immunity in vulnerability to impulse-control disorders.

## Introduction

Besides the core symptoms of hyperactivity, impulsivity and impaired sustained attention, which are also found in other syndromes, children with attention-deficit hyperactivity disorder (ADHD) often display accompanying socio-behavioural difficulties, including defiant-opponent symptoms and disinhibited conduct [1]. According to the dominant model, ADHD is viewed as an executive dysfunction [2,3], but alternative accounts present ADHD as a motivational dysfunction [4], arising from altered processes within fronto-striatal circuits [5,6]. Frequently comorbid with ADHD, obsessive-compulsive disorder (OCD) is a chronic, progressive disorder with a prevalence of 1-4%. OCD is essentially an impulse-control disorder, commonly comorbid to symptoms like compulsive shopping and/or sex, pathological gambling, Tourette's syndrome [7,8], and may also be conceptualized as part of the addictive disorder spectrum [9]. In fact, more than half of ADHD patients have an obsessive-compulsive, schizotypal and paranoid personality, as well as substance abuse / dependence problems [10,11].

In both ADHD and OCD, a lack of self-control capacities may provide the ground for involvement in extreme and risky activities, such as those typical of the sensation-seeking supertrait [12]. There is substantial genetic influence in impulse-control disorders: for instance, the dopamine D4 receptor (DRD4) is the prototypic polymorphic gene, subserving a background for novelty- and risk- seeking [13,14], addiction [15] as well as both ADHD and OCD [16-18]. Among studies describing alteration of dopamine systems in ADHD and/or OCD, it has been proposed that specific symptoms may arise from a modification in dopamine transporter (DAT) expression and function [19-22]. In this line, a rising interest exists for auto-immune processes and psycho-immunological interactions (see e.g. [23-25]) which - among other non-genetic factors that obviously play a considerable role - may account for such possible DAT alterations.

Although the blood–brain barrier (BBB) forms a tight seal that might impede circulating molecules entering into the CNS [26,27], the immune system appears to have no mechanism to prevent the production of antibodies against brain antigens, making it likely that there could be an antibody-centred immune response in the context of a BBB breakdown [28,29]. A breach in BBB integrity can be caused under conditions of stress [30], or owing to a traumatic injury to the brain (TBI) like e.g. as complication of a difficult delivery [31]. Once admitted the possibility of a BBB failure, implications include drainage of CNS antigens to peripheral lymphoid organs, with subsequent auto-immune response towards the CNS: these potentially self-directed antibodies (i.e. auto-antibodies, aAbs) may recognize a wide range of CNS proteins, including AMPA and NMDA glutamate receptors [32]. Conversely, whenever the integrity of BBB is compromised, different molecules including aAbs can freely reach the CNS.

Besides Systemic Lupus Erythematosus (SLE), a prototypical disease caused by anti-nuclear antibodies [33], neuron-binding aAbs have been detected in sera from several patients: aAbs to glutamate receptors have been evidenced in intractable seizures [34,35] or brain ischemic stroke [36]. In the context of neuro-psychiatry, autoimmune responses are common in Hashimoto’s encephalopathy, Sydenham’s chorea, chronic opiate addictions [37], as well as OCD and schizophrenia [25]. Interestingly, similarly to other pediatric autoimmune neuropsychiatric disorders associated with streptococcal infection (PANDAS), the development of Tourette’s syndrome in children and in adolescents [38,39] has also been ascribed to anti-neuronal antibodies. Thus, symptoms of ADHD may partially share some pathogenesis’ steps, if not etiology, with Tourette’s and/or OCD: in that some symptoms may be proposed as a neuro-psychiatric sequel of a streptococcal infection [23,24,40].

Circulating aAbs to several CNS antigens can be detected in experimental animal models [41-46], as well as in opiate-treated mice [47]. Presently, we have hypothesized that anti-DAT aAbs could cross the BBB, reach DAT proteins on neuronal synapses, and hence produce reactions culminating in a long-term functional interference over these neurons. Although presently unable to cover all steps of such hypothesis, as a preliminary study we generated an auto-immune response possibly interfering with DAT function, and then tested in-vivo the long-term consequences. In particular, mice were immunized with two synthetic fragments, whose design was based on DAT protein sequence, to generate a rise in DAT-targeting aAbs. Such auto-immune challenge could in turn lead to an enduring and possibly detectable interference with the dopamine (DA) neuro-transmission as well as to DA-related behavioural changes. Aim of and rationale for this investigation was to test whether some ADHD and/or OCD behavioural symptoms, like impulsive and/or compulsive behaviour, may be generated in-vivo via an auto-immune challenge.

## Methods

All procedures were approved by the local Animal Survey Committee, on behalf of Italian Ministry of Health, and were carried out in accordance with the European Community Council directive (86/609/EEC) and Italian Law. All efforts were made to minimize animal suffering, to reduce the number of animals used, and to use alternatives to in-vivo testing.

### Animals and immunization

Male CD-1 mice (Charles River, Calco, Italy), at weaning age (21 days old, PND 21) upon arrival, were housed in pairs within Plexiglas cages (33 × 13 × 14 cm) and kept at constant temperature (22 ± 1°C), upon a reversed light/dark cycle (lights on from 20.00 to 08.00). Food (Altromin-R, A. Rieper S.p.A., Vandoies, Italy) and water were available *ad libitum*.

**Scheme 1:**
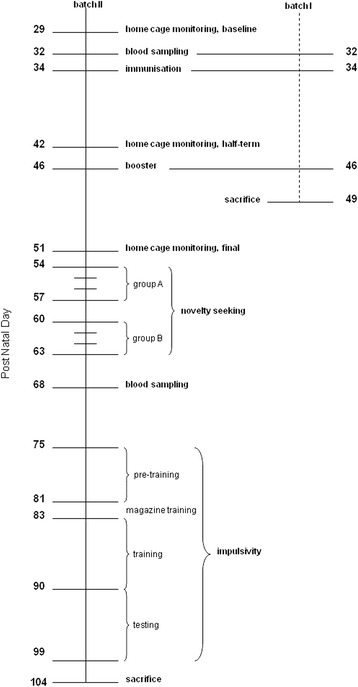
Experimental design, showing mice in batch I (N = 24) and batch II (N = 24).

The experiment was run in two batches of mice (see Experimental Scheme 1): mice of batch I (N = 24) were sacrificed on PND 49, after immunization and boost, to verify the generation of circulating DAT aAbs; mice of batch II (N = 24) underwent a test battery, to evaluate long-term changes in behaviour after immunization and boost. Following arrival of each batch, after seven days of acclimation, mice were randomly assigned to two groups, i.e. DAT-immunized (DAT-i) or vehicle controls (VEH, n = 10–14 per group). DAT-i mice were injected intraperitoneally (i.p.) with Freund’s Adjuvant vehicle, both the Complete (CFA, immunization at PND 34) and then the Incomplete (IFA, boost at PND 46). Plasma levels of DAT aAbs were monitored in mice of both batches, to prove effective immunization. Body-weight of mice was monitored in batch II, on PND 32, 68, 76 and daily during the delay-of-reward task (PND 83–99), to detect any possible effect on bodyweight gain, due to or following immunization.

The immunogen was formed by peptide fragments, designed by one of us (O.G.) by selecting a 19-aminoacid portion of the DAT sequence based on the most immuno-reactive portion (U.S. Patent and Trademark Office: EFS ID 13464574; Application Number: 61681638; Granstrem et al. 10-AUG-2012 provisional patent), and custom synthesized (DiaPharm Ltd. Russia). Such immunogen was used within CFA or IFA emulsion for immunization and boost. For each animal, the immunogen (60 μg) was dissolved in 0.125 ml sterile saline and prepared by adding 0.125 ml CFA or IFA, to form a stable emulsion [48]. Control mice received a VEH treatment without any immunogen, i.e. an emulsion of sterile saline plus either CFA (at PND 34) or IFA (at PND 46).

### Blood samples, aAbs detection

All animals were withdrawn around 100–200 μl of blood as a baseline sample (on PND 32) by lateral tail vein incision. The animals were not anesthetised nor constricted: they were gently allowed to move semi-freely on the cage-top grid, while the experimenter was holding the tail to proceed with the incision (using a blade) and with blood collection (using a sampling vial), then left to freely-moving recovery over a cage-top grid for 10 sec. For mice of batch I, blood was collected at sacrifice (PND 49, i.e. three days after boost). Mice anesthetised with xilazine and ketamine (10:100 mg/kg) were bled by intra-cardiac puncture. For mice of batch II, blood was obtained at PND 68, i.e. three weeks after the boost, by lateral tail vein incision (as above). Samples were centrifuged at 3,500 rpm for 20 min, and plasma was stored at −80°C until subsequent analysis. In this way, each mouse provided two samples differing for “timing” of withdrawal (i.e., one baseline sample at PND 32 and one after-boost sample, at PND 49 in batch I and PND 68 in batch II). The difference in “end sampling” between the batches (i.e., batch I = 3 *days* after-boost *versus* batch II = 3 *weeks* after-boost) allowed to test 1) whether the immunization was effectively generating DAT aAbs (in batch I), and 2) whether this phenomenon recovered long after the boost (in batch II).

Peptide fragments of DAT were used as antigens, to detect plasma aAbs levels by means of a modified ELISA [41,42]. In brief, the plasma samples (diluted 1:20,000) were applied (100 μl) on the immuno-plate (Costar, USA) containing the corresponding peptide fragment (0.5 μg/well) and blocking agent. The plate was incubated (1 h, 25°C) and washed (PBS with 0.05% Tween 20, pH 7.4). Peroxidase-labeled rabbit anti-mouse-Ig (diluted 1:1000) secondary antibodies (Sigma, USA) were added (100 μl) to each well and incubated (1 h, 25°C). The plate was washed for 15 min with PBS and then the substrate solution, o-phenylen-diamine SIGMA FAST™ (Sigma, USA), was added (100 μl) to each well. The reaction was terminated by adding 2 N H_2_SO_4_, and the plate was scanned at 450 nm on a Microplate Reader 3550 (Bio-Rad, USA). Sample buffer was included as a blank, its value being subtracted from all samples (measured in triplicate). This ELISA-based kit cannot discriminate among the specific sub-classes or typologies of aAb.

### Monitoring home-cage activity

Mice of batch II were monitored for spontaneous home-cage activity [49]. This was done thrice: 1, five days before immunization (baseline, PND 29–31); 2, after immunization (PND 42–44); 3, five days after the boost (PND 51–53). An automatic device was used, with small passive infrared sensors on the top of each cage (Activiscope, Techno-Smart, Roma, Italy; http://www.newbehavior.com), which detected any movement of mice (sampling rate 20 events per second, 20 Hz). Data, recorded by a computer with dedicated software, consist of cumulative scores obtained during 60-min intervals, expressed as counts per minute (cpm). Then, a circadian profile (24 points, 1 h each) of activity was obtained by averaging three consecutive days of continuous registration within individual cages. Spontaneous home-cage activity counts were analyzed separately for diurnal and nocturnal phases, which were further divided in early four-hour and late eight-hour portions. The access of authorized personnel to animal room was not restricted and followed the routine schedule.

### Novelty-seeking test

#### Apparatus

The experimental apparatus consisted of an opaque Plexiglas rectangular box with smooth walls and floors, which was subdivided into two compartments (20x14x27cm each). The opening between the two compartments could be closed with a temporary partition. Visual cues were associated with each compartment: one compartment had a white floor, one white wall and three black walls, while the other compartment had a black floor, one black wall and three white walls. Each compartment was provided with four pairs of infrared photobeams, placed on the wall at a few cm above the floor, spaced 5.5 cm apart. Beam interruptions were recorded by a computer with custom-made software. The following data were obtained automatically: 1) time spent in each compartment, 2) activity rate in each compartment (number of beam interruptions/second), and 3) frequency of crossings between the two compartments (number of crossings/minute). The whole session was subdivided into bins, i.e. partial 5-min intervals. Two boxes placed in a soundproof test room with dim illumination were used; each subject was always tested in the same box. The floor of the test apparatus was cleaned after each animal.

#### Procedure

The experimental schedule (see ref. [50]) took a total of four days, each subject (of batch II) being tested either at PND 54–57 (one cage mate) or at PND 60–63 (the other cage mate) between 13.00 and 17.00. Testing of different experimental groups was counter-balanced across time. During testing, the remaining cage mate stayed in the animal room to prevent visual, auditory or olfactory communication between cage mates. On the first three days, mice were placed in the black floor/white wall compartment of the apparatus for 25 min, in order to familiarise with the procedure and apparatus, while the other compartment was left unknown. On the fourth day, animals were placed in the familiar compartment as usual. Then, the partition separating the two compartments of the apparatus was opened after 20 min, and mice were allowed to freely explore both compartments of the apparatus for 15 min.

### Delay-of-reward task

#### Apparatus

Four computer-controlled operant chambers (LabLink, Coulbourn Instruments, Allentown, PA, USA) were used. The chambers, made of aluminium and plexiglas with grid floor, were provided with two nose-poking holes, two feeder devices, two magazines (placed above the nose-poking holes) where precision pellets (20 mg, BioServ, Frenchtown, NJ, USA) were dropped, two magazine lights, two chamber lights (one over each magazine), and an aluminium platform designed to make the food magazines accessible to mice by ramp climbing. Nose-poking into the holes was detected by a photocell and was recorded by a computer with custom-made software, which also controlled food delivery. Each mouse was tested daily in the same chamber. The grid and walls of the chamber were cleaned after each animal.

#### Procedure

Before the schedule started, animals (batch II) now at adulthood (PND > 75) were familiarised with the precision (BioServ) pellets, provided in their home cage on three successive days. Subsequently, each animal was individually placed in the operant chamber for 20 min daily. After this session, mice were returned to their home cages, where they were given standard food (approximately 65% of their *ad libitum* food intake), in order to restrict them up to 90 ± 5% of their free-feeding bodyweight. This procedure resembles the mild level of food restriction, used in other studies [51-53] on decision making in rats and mice. Food restriction was intended to increase animal’s motivation to work for food delivery during training and testing phase.

The first day was just for familiarisation with the novel environment (no pellets delivered). On the subsequent day, the “training” phase started (8 days), providing binary choice between two alternatives. Now, nose-poking in one of the two holes, termed small-and-soon (SS) hole, resulted in the delivery of one pellet of food in one magazine, whereas nose-poking in the other hole, termed large-and-late (LL) hole, resulted in the delivery of five pellets in the other magazine. After nose-poking and before food delivery, the chamber light on the nose-poked side was switched on for a 1-sec flash. After food delivery on the nose-poked side, the corresponding magazine light was switched on for 25 sec (time out, TO), during which any additional nose-poking was without any scheduled consequence. Mice were not trained to a fixed criterion, rather they were provided a fixed number (i.e., eight) of daily sessions, which enabled the development of a reliable LL preference in nearly all of animals (see ref. [50]).

During the “testing phase” (9 days), we evaluated (in)tolerance to large-reward delay. Now, a delay was inserted between nose-poking in the LL hole and the delivery of the five-pellet reward. The chamber light on the LL side was switched on during the whole length of this delay. Any additional nose pokes during this interval were ineffective, i.e. not reinforced (“inadequate responding”, [5,54]). The contingencies of the SS reward, obtained by nose-poking at the SS side, were unchanged. Under these conditions, nearly half of animals are expected to shift in preference from LL to SS (“steep” sub-population) while the other half maintain a clear-cut preference for LL (“flat” sub-population, see [55]).

The whole experimental schedule took 20 days; subjects were tested between 10.00 and 17.00. The delay length was kept fixed for each daily session and was progressively increased across subsequent days (0, 5, 10, 17, 30, 42, 60, 90, 120, 150 sec). To compare present results with similar studies, delay values should be converted into odds [56]. The “relative” impact of present delays did therefore follow this sequence: 0, 0.07, 0.14, 0.24, 0.42, 0.58, 0.83, 1.25, 1.66, 2.08 delay-equivalent odds. The dependent variables were: 1) the choice (%) for the large reinforcer, i.e. the percentage of preference for LL over total choices; and 2) the “slope” of the preference-delay curve. For each individual mouse, the slope value was calculated using the Microsoft Excel “slope” function, with LL-preference as the *y*-axis data and log(delay + 1) as the *x*-axis data [57].

### Monoamine measurement ex-vivo

Five days after the delay-of-reward task, at 3.5 months of age, mice (batch II) were sacrificed; brains were quickly removed, dissected on dry ice and stored at −80°C. Concentrations of serotonin (5-hydroxy-triptamine, 5-HT), dopamine (DA) and their metabolites, 5-hydroxy-indole-acetic acid (5-HIAA), 3,4-dihydroxy-phenyl-acetic acid (DOPAC) and homo-vanillic acid (HVA), were then assessed in the prefrontal cortex (PFC), striatum (whole complex, including both dorsal and ventral portions). Indeed, the behavioural flexibility assessed by the delay-of-reward task is well known to depend on the interplay between PFC and striatum [58-64].

Quantification of these monoamines, expressed as ng/dg wet tissue, was carried out using a modified version of the classical high performance liquid chromatography, combined with an electrochemical detector (HPLC-EC), as described by others [65,66]. These data provide information about function of forebrain neuro-transmitter systems.

### Statistical analyses

#### General ANOVA

To detect differences due to the immunization procedures on the data collected as mentioned above, repeated-measure ANOVAs were performed (Statview II, Abacus Concepts, USA), with “Immunization” as a between-subjects factor (namely, VEH *versus* DAT-i) and “Time” as a within-subject factor (namely, the 24-level scores, one per hour; or the 3-, 4-, or 5-level bins, one per 5-min partial interval). Additional factors were introduced when required, as outlined below. Multiple comparisons were performed for all parameters by the post-hoc Tukey HSD test, an independent statistical source: significant effects are drawn whenever differences between means are apparently beyond the Tukey HSD threshold [67]. Statistical significance was set at *p* ≤ 0.05 (two-tailed).

#### ELISA-based aAbs detection

A 2x2x2 split-plot ANOVA was performed, by adding a factor accounting directly for two separate batches. Therefore, a between-subjects factor termed “Batch” (namely, date of second withdrawal from either batch: PND 49, 3 days after boost *versus* PND 68, 3 weeks after boost) was added to the general design, where “Immunization” (VEH *versus* DAT-i) was a between-subjects factors and “Timing” (of withdrawal from the same animal: baseline *versus* after-boost) was a within-subject factor. Separate ANOVAs were performed within either level of a given factor when allowed.

#### Subgroup analysis

According to a procedure which has been widely adopted and validated in a number of studies [55,68], each immunization group was divided into two subgroups, on the basis of slope in the preference-delay curves. Mice with lower slope values (in algebraic value) were assigned to one subgroup; mice with higher slope values (in algebraic value) were assigned to the other subgroup.

#### Delay-of-reward task

After the formation of subgroups, data were re-analyzed. Selection of lower- *versus* higher- slope values resulted in the segregation (to either subgroup) of mice within each pair of cage-mates, suggesting that mice within each pair were statistically not independent. Therefore, the pair of (non-independent) cage mates was the statistical blocking factor: a within-pair factor termed “Subgroup” (namely, “low-slope” *versus* “high-slope” as defined above) was added to the design, where “Delay” (9-level daily session, 0-150 sec) was a within-pair factor, and “Immunization” (VEH *versus* DAT-i) was a between-pairs factor. The design was a 2x2x2 split-plot ANOVA.

#### Monoamine measurement

After the formation of subgroups, data were re-analyzed by a two-way split-plot ANOVA, with a 2x2 design. The within-pair factor termed “Subgroup” (“low-slope” *versus* “high-slope” as defined above) was added to the between-pairs “Immunization” (VEH *versus* DAT-i) factor.

## Results

### Generation of circulating anti-DAT aAbs, body-weight

The efficacy of immunization was assessed by measuring plasma levels of aAbs targeting the DAT fragments, by using a modified ELISA test (see Table 1). Accordingly, after immunization with the antigen in CFA and boosting with the antigen in IFA, the plasma aAbs levels of all mice (i.e., both groups in both batches) were significantly elevated, compared to the baseline condition (Timing, F(1.95) = 11,12, *p* <0.05). Noteworthy, results clearly indicate an immunizing effect of the DAT fragments (Batch x Immunization, F(1,95) = 5.33, *p* <0.05). Indeed, increase of DAT-aAbs titers was particularly marked in DAT-i mice of batch I (six-fold over VEH controls).

**Table 1:** Circulating aAbs against DAT

**Batch** : **2**^**nd **^**withdrawal**	**3 days after boost**	**3 weeks after boost**
VEH controls	385 ± 118%	157 ± 140%
DAT-i mice	2248 ± 866% **	156 ± 108%

Mean (±SEM, n = 12 per group) percent increase in plasma levels of DAT-aAbs titers in the second withdrawal compared to baseline, as a function of immunization group. Each mouse provided two samples differing for “timing” of withdrawal (i.e., baseline sample at PND 32 *versus* a second after-boost sample). The “batches” differed (see Experimental Scheme 1) for the second withdrawal, which was taken at PND 49 in batch I and PND 68 in batch II (i.e., 3 days after boost *versus* 3 weeks after boost). ** *p* < 0.01 between DAT-i and VEH control group.

As expected, plasma levels of DAT aAbs in all mice (i.e., both VEH and DAT-i) were significantly higher in samples taken a few days after the boost (in batch I), compared to samples taken three weeks later (in batch II), when they also became indistinguishable between VEH and DAT-i groups (Batch, F(1,95) = 4.28, *p* <0.05). Thus, as is classically the case, DAT-aAbs titers completely recovered by three weeks [69]. Post-hocs confirmed the profile just described, with a significant, six-fold difference between the DAT-i and the control group (*p* <0.01) in peak titers, observed in batch I a few days after the boost. This difference was not found in samples from batch II, taken three weeks after the boost. Therefore, two separate analyses were carried out within the immunization factor.

Within the DAT-i group, significance emerged for batch (F(1,53) = 3.83, *p* <0.05), timing (F(1,53) = 7.29, *p* <0.05), and their interaction (Batch x Timing, F(1,53) = 3.16, *p* <0.05). DAT-fragment immunization significantly elevated DAT aAbs titers, twenty-fold compared to baseline. These levels reached peak values in batch I, few days following the boost. In batch II, where the second sample was taken three weeks later, aAbs levels were still slightly elevated over the baseline, but markedly recovered in comparison to batch I. As for the control group, plasma aAbs levels were slightly but significantly elevated by VEH alone (Timing, F(1,42) = 4.49, *p* <0.05). This represents a non-specific stimulation (by CFA and IFA), found for the few days following the boost, with slight and non-significant recovery afterwards. Such a profile of results serves to demonstrate indeed that the immunization procedure was fully able to stimulate a reaction by the immune system. A very small (three-fold) increase of DAT aAbs was transiently generated also in the absence of the antigen in VEH controls, while the presence of the peptide immunogen in DAT-i mice clearly led to strong anti-DAT titers.

No difference in body-weight was produced in batch II by the immunization procedure.

### Peaks of activity in circadian rhythm

There were no significant group differences in the baseline as well as the intermediate recording periods, taken before first immunization and before the boost. Significant effects emerged for the last recording period, taken few days after the boost (at PND 51–53), but only when analyzing separately the two activity peaks usually observed in mouse circadian rhythm. Specifically, the early four-hour portions of both diurnal and nocturnal phases underwent ANOVA with a 4-level rather than 24-level "Time" factor as a repeated measure; findings were then confirmed by Student’s T tests performed on total portion’s score. After offset (9 h-12 h) of room light, we found a main immunization effect in the ANOVA, F(1,10) = 4.67, corresponding to t(10) = 2.16, .05 < p < .10, for the nocturnal peak; after onset (21 h-24 h) of room light, we found a main immunization effect in the ANOVA, F(1,10) = 3.31, corresponding to t(10) = 1.82, .05 < p < .10, for the diurnal peak. The activity full (24 h) profile was then screened with Tukey HSD.

**Figure 1:**
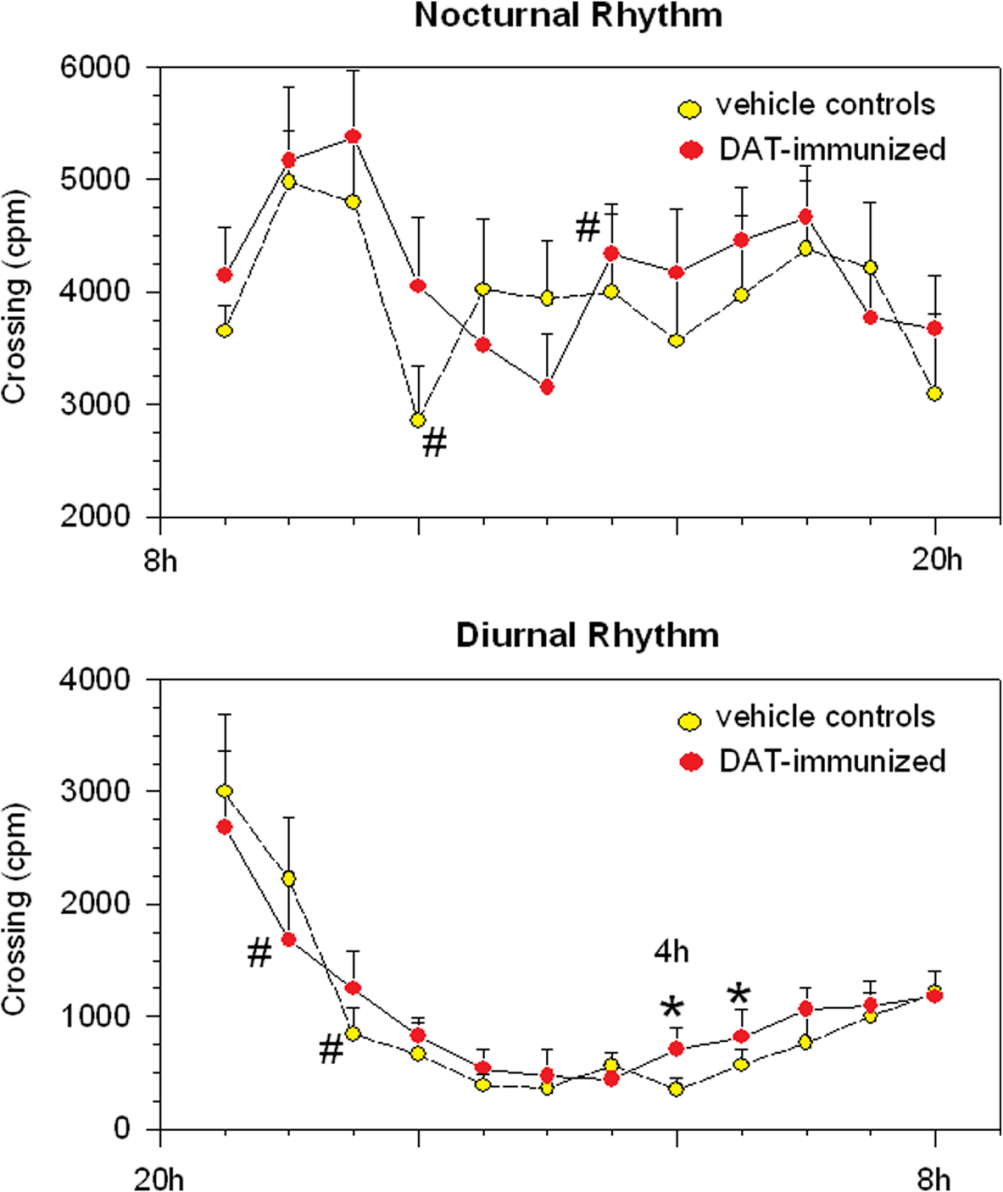
**Circadian Rhythm of Spontaneous Activity. **Mean (±SEM, n = 6 pairs) counts per minute (cpm) by undisturbed mice, housed in pairs per home cage (sensor rate 20 Hz). Nocturnal (upper panel) and diurnal (lower panel) activity was recorded for 3 days (PND 51–53), five days after the boost with DAT fragment (DAT-i) or VEH. The 24-h profile of one-hour points was then obtained by averaging the 3 days of continuous registration. * *p* < 0.05 between DAT-i and VEH control group; # *p* < 0.05, compared to the previous or the following 1-h point within the same immunization group.

At light-offset, VEH-injected controls showed peak activity followed by a sharp decrease afterwards, as witnessed by a clearly significant difference found between 11 h and 12 h (see Figure 1, # symbol). Interestingly, after light-offset, DAT-i mice displayed a prolonged peak of activity, with no evidence of such a sharp decrease: rather, activity levels were showing a slight decrease continuing well after 12 h. Indeed, a relative minimum for DAT-i mice was found at 14 h, followed by a sharp and significant increase between 14 h and 15 h (see # symbol). Compared to the profile of VEH mice, data suggest a 2 h-longer peak at wake-up, with the relative minimum of activity being shifted onward (from 12 h to 14 h) in DAT-i ones.

Moreover, VEH-injected controls showed a sharp decrease of activity after light-onset, as witnessed by a clear-cut and significant difference found between 22 h and 23 h (see # symbol). Such a profile was anticipated in DAT-i mice, occurring 1 h earlier between 21 h and 22 h. Compared to VEH mice, therefore, the starting point in resting phase of circadian activity was anticipated (from 22 h to 21 h) in DAT-i ones. However, the Tukey HSD suggested for DAT-i mice a slight elevation of spontaneous activity at 4 h and 5 h (see Figure 1, * symbol), namely in the very middle of their resting period.

In brief, DAT-fragment immunization produced: 1) a 2 h-longer peak of activity seen at light-offset; 2) a 1 h-anticipated beginning of rest after nocturnal daily activity; and 3) a wake-up attempt during the middle of the daylight.

### Reactions to novel environments

The ANOVA on data from the first day of exposure to the novel environment yielded an effect of time, F(4,88) = 75.4, *p* < .001, but not of immunization, F(1,22) = 2.94, p > .10, NS. Activity levels were indistinguishable both after 5 min of exposure, suggesting a similar novelty-induced peak (2.7 ± 0.2 in average for both groups), and after 25 min of exposure (1.5 ± 0.1 in average for both groups), suggesting a similar habituation to novelty.

The ANOVAs on the testing day did not yield significance for immunization effects nor for their interaction with time. As a matter of fact, the two groups did not differ in time spent in the novel side, activity in either side, nor crossings between sides (see Table 2).

**Table 2:** **Reaction to novelty in VEH and DAT**-**i mice**, **in a novelty**-**seeking test**

**Parameter**	**Activity rate**	**Time (%) in novel**	**Crossing**/**min**
VEH controls	3.79 ± 0.21	79.7 ± 1.09	2.55 ± 0.26
DAT-i mice	4.10 ± 0.22	78.6 ± 1.75	2.98 ± 0.33

After a three-day familiarization to one side, mice had free-choice access to a novel side of the apparatus. Mean (±SEM, n = 12 per group) activity rate (number of beam interruptions per second), time (%) spent in the novel side, and frequency of crossing to the familiar side, measured via photo-beam interruptions by mice on the testing day.

### Choice behaviour with delayed reward

**Figure 2:**
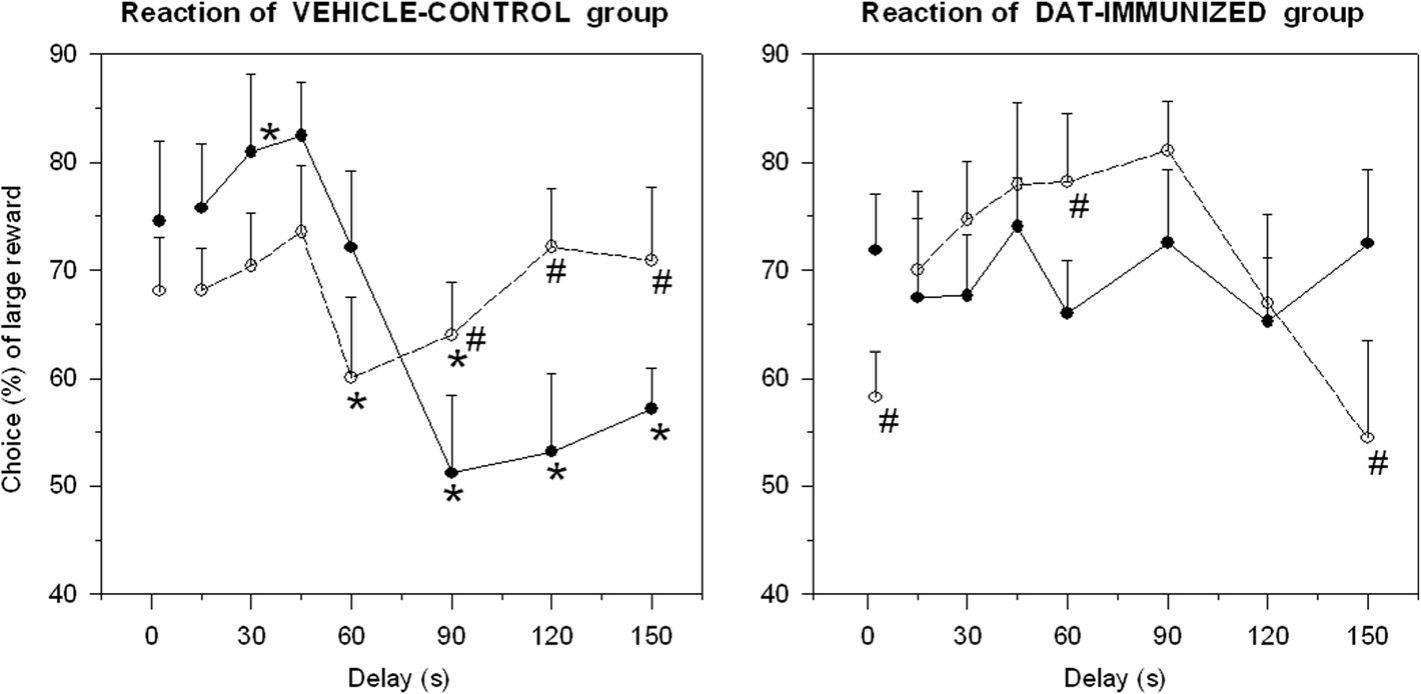
**Choice between Rewards**, **Reaction to Delay. **Mean (±SEM, n = 6 per sub-population) choice (%) for the large but late (LL) reward in mice, immunized with DAT fragment (right panel) or vehicle alone (left panel), in a delay-of-reward task. Two sub-populations were formed, based on the slope of preference curves (see Methods). Among VEH controls, individuals segregated into either a flexible (with a “steep” profile, black points) or an inflexible (with a “flat” profile, white points) subgroup. Among DAT-i mice, individuals segregated into either an inflexible (with a “stuck” profile, black points) or a flexible (with a “slow” profile, white points) subgroup. * *p* < .05 between DAT-i and VEH controls; # *p* < 0.05 when comparing sub-populations (low- *versus* high-slope) within the same immunization group.

ANOVA revealed a trend for delay, F(9,90) = 1.64, .05 < *p* < .10, delay by immunization, F(9,90) = 1.61, .05 < *p* < .10, and significance for delay by immunization by subgroup, F(9,90) = 1.88, *p* < .05. Such a complex pattern of results was first investigated with post-hoc analyses, aimed at comparing the two subgroups (see # symbols in Figure 2). Within the VEH controls, a significant difference between subgroups appeared only for delays of 90s, 120 s, 150 s. At these points, classical “flat” and “steep” profiles emerged (Figure 2, left panel), as expected [55]: the former subgroup did not abandon its original preference for LL, whereas the latter exhibited a prominent shift towards SS, whereby their preference eventually stabilized around chance level.

In clear contrast, within the DAT-i mice, the lower-slope subjects (termed “inflexible”) were characterized by not showing any steepness at all (Figure 2, right panel): this subgroup was just fluctuating between 65% and 75%, suggesting a rigid or stuck preference. Conversely, the higher-slope subjects turned out to be characterized by a marked upward steepness (i.e., “flexible” but in the opposite direction): this subgroup started with a scarce performance at 0 s, and then displayed progressively more choices for LL options until a peak was reached at 60s. Noteworthy, these peculiar positive-slope mice were apparently slow in developing a preference for LL; they eventually showed some indication of temporal discounting, but only at the longest delay interval (150 s). Such kind of behavioural profiles shall not be interpreted as an index of impulsivity, but rather in terms of choice flexibility. This profile of findings was confirmed when separate analyses were run for either sub-population, followed by post-hoc analyses aimed at comparing DAT-i to VEH mice (see * symbols in Figure 2).

The two sub-populations with higher slope values (open circles in Figure 2) comprised “flat” VEH *versus* “slow” (flexible) DAT-i mice. The ANOVA did not yield significance for delay effects, nor for the interaction with immunization. However, a significant difference emerged at delays of 60s and 90s, with “flat” VEH mice showing lower LL preference than “slow” (flexible) DAT-i mice. This finding indicates that - although less flexible - subjects among VEH controls with a “flat” profile were indeed capable of reacting to delay intervals (i.e., by showing a transient shift towards SS at 60s and 90s then followed by a robust recovery in the expression of LL preference). Conversely, at delays of 60s and 90s, the “slow” (flexible) DAT-i subjects were still developing initial LL preference. Compared to VEH mice, a sign of reaction to delay was only seen at 150 s (not at 60s), thus confirming a flexible but very slow reactivity to task contingencies in these DAT-i mice.

The two sub-populations with lower slope values (closed circles in Figure 2) comprised “steep” VEH *versus* “stuck” (inflexible) DAT-i mice. ANOVA yielded significance for delay, F(9,90) = 1.86, *p* < .05, and for the interaction with immunization, F(9,90) = 1.91, *p* < .05. Post-hoc comparisons revealed a significant difference between “steep” VEH and “stuck” DAT-i mice at delays of 30s (with a higher LL preference in the former) and again at 90s, 120 s, 150 s (with a lower LL preference in the former). Data confirmed that flexible subjects among VEH controls (i.e., those with a “steep” profile) developed (for delay > 60s) a robust shift due to a temporal discounting, which stabilized around chance level namely with choosing SS and LL equally often. Conversely, the sub-population of “stuck” (inflexible) DAT-i mice did not show any contingency-driven change nor any transient attraction towards either LL or SS. Their apparent performance just fluctuated around an average 70% of LL preference.

Peculiar performance of inflexible DAT-i mice (with a “stuck” profile) was confirmed by data collected during the training phase: number of sessions with a significant preference for LL differed across subgroups. These values were indistinguishable between less *versus* more flexible VEH sub-populations (3.5 ± 0.8 days in both subgroups), suggesting a similar development of LL preference. Conversely, this parameter was significantly higher in inflexible DAT-i mice than in flexible ones (5.5 ± 0.8 *versus* 2.0 ± 0.4 days, respectively, when comparing the two profiles: “stuck” *versus* “slow”). These data suggest somewhat an earlier manifestation of LL preference in the former subgroup. Most of the inflexible DAT-i mice (with a “stuck” profile) displayed LL preference by the third training session (which for mice is surprisingly too quick, see ref. [70]), and then maintained it throughout the entire task.

In summary, VEH mice exhibited the well expected and clear-cut reaction to the increasing delay. Individual differences emerged, with one sub-population showing inflexible choice (i.e., actively choosing to maintain a LL preference, after a transient attraction towards SS) and the other one showing flexible delay-intolerance (i.e., quickly shifting from LL preference towards SS). In clear contrast, performance of DAT-i mice was quite impaired. Half of subjects were inflexible (i.e., stuck to a rigid choice habit despite a progressively increasing delay); the other half of mice were just residually flexible (i.e., considerably slow to acquire and to shift their choice strategies).

### Forebrain monoamine impairment

#### DA levels

No overall difference was found between DAT-i and VEH mice in the striatal levels of DA and of DOPAC, the major metabolite of DA (see Table 3). However, when considering the classification into two sub-populations, which were evidenced at behavioural level, marked differences appeared (immunization x subgroup, F(1, 20) = 4.52, *p* < 0.05). Namely, within the DAT-i group, a significantly higher DOPAC concentration was found in the striatum of inflexible mice (with a “stuck” profile, see black circles, Figure 2) compared to flexible ones (with a “slow” profile, see white circles, Figure 2). Overall, DAT-i mice showed significantly higher striatal levels of HVA than VEH mice (immunization, F(1, 20) = 6.86, *p* < 0.05).

**Table 3:** **Neuro**-**chemical parameters in striata of VEH and DAT**-**i mice**

**Parameter**	**DA**	**DOPAC**	**HVA**
VEH inflexible (“flat”)	317.5 ± 47.5	91.3 ± 22.0	59.5 ± 5.7
VEH flexible (“steep”)	368.5 ± 35.9	77.2 ± 20.0	65.4 ± 2.5
DAT-i flexible (“slow”)	320.8 ± 30.4	53.8 ± 14.9	75.2 ± 9.7 *
DAT-i inflexible (“stuck”)	452.3 ± 90.9	124.9 ± 22.2 *#	88.3 ± 9.2 *

Mean (±SEM, n = 6 per sub-population, N = 24) concentration (ng/dg wet tissue) of striatal DA and its metabolites, as a function of immunization group and of the sub-population as emerged in the operant-behaviour task. * *p* < 0.05 between DAT-i and VEH control group within the corresponding slope-defined sub-population; # *p* < 0.05 between sub-populations within the same immunization group.

Considering the PFC, no effect of immunization emerged. However, a significant difference emerged between the two separate sub-populations (subgroup, F(1,20) = 7.24, *p* < .01). Indeed, the lower-slope subgroup (formed by mice with a “steep” or a “stuck” profile, see black circles, Figure 2) had higher levels of HVA (10.49 ± 0.79 vs 7.97 ± 0.45) than the higher-slope subgroup (consisting of mice with a “flat” or a “slow” profile, see white circles, Figure 2). These data may suggest a better cortical function in the latter subgroup of subjects. Note that mice with a “flat” or a “slow” profile do share a common feature, that is, they display as a whole a more marked attraction to LL, compared to mice with a “steep” or a “stuck” profile.

#### 5-HT levels

DAT-i mice exhibited significantly higher levels of 5-HT in the striatum (33.06 ± 2.19 vs 25.71 ± 2.82), when compared to the VEH controls (immunization, F(1, 20) = 4.43, *p* < 0.05). The elevation in 5-HT levels may compensate for the striatal DA impairment in DAT-i subjects. No effects whatsoever were found in the PFC.

## Discussion

Immunization with DAT fragments generated a transient auto-immune response, with a marked elevation in titers of circulating aAbs directed against DAT. Notably, this response resulted in long-lasting impairment of striatal DA systems’ function, with enduring rise of HVA and also DOPAC levels. Such neuro-chemical sequel was consistently associated with a profile of behavioural modifications, including a rearrangement in patterns of nocturnal activity and diurnal resting, spontaneously expressed within home-cages settings. Such subtle effects in the wake-sleep rhythm were expected based on earlier data [49,55]: these effects are relevant, since they denote a longer and more intense beginning as well as an earlier end of the daily spontaneous activity in DAT-i mice. Interestingly, a considerable impairment of their adaptive capacity was found when considering choice behaviour in the delay-of-reward task contingencies.

### Changes in behavioural flexibility and impairment in forebrain DA

In a classical study from our group [55], we assessed spontaneous home-cage activity in the SHR rat, a validated model for ADHD [54]. Interestingly, these rats showed increased activity during specific time-points of the dark phase, as well as peaks of activity with interruption of rest during the light phase. Thus, the circadian rhythm of present DAT-i mice, consisting in a prolonged peak of spontaneous activity after wake-up, an anticipated start for the resting period, as well as the tendency for diurnal sleep to be interrupted by a wake-up peak, shares some interesting features with these rats modelling for ADHD.

As for operant-choice behaviour, control mice were able to perform the whole delay-of-reward task correctly. As expected, all of the subjects developed a clear LL preference during training phase and then modified their choice preference according to the introduction of delays. Indeed, they displayed either a slight and transient or a robust and persistent shift towards SS, denoting their subjective discounting of value attributed to the LL option. As classically reported with this kind of protocols [57,71], discounting (or not) of LL value identifies these individuals as more (or less) delay-intolerant.

Notably, mice of the DAT-i group appeared greatly impaired in operant choice between two alternatives differing for reward size and delay contingencies. Noteworthy, they were perfectly capable to associate nose-poking in either hole with the corresponding magazine of food-delivery. Nose-poking was followed by ramp climbing and food eating, ruling out major biases due to gross motoric or cognitive deficit. Their impairment was apparently specific to the formation and/or the flexible modification of a choice strategy. In fact, half of the subjects (termed DAT-i flexible, with a “slow” profile) were hardly able 1) to discriminate the difference in reward size between LL and SS, and 2) to detect and/or react to delay intervals. These subjects were still flexible, but appeared to be slow both in developing preference for the larger-size reward and in reacting to its delay; this, despite extensive training and testing. In the other half of subjects (termed DAT-i inflexible, with a “stuck” profile), a more marked impairment emerged: a net preference for LL seemed to be expressed, which was likely the result of a rigid pattern of choice. Closer analyses on sequence of choices may be useful to ascertain if this was indeed a habit (informal observation of mice suggested that each SS option was followed by two LL options quite regularly).

Present DAT-i mice displayed two profiles, consisting of either slower acquisition of LL preference plus late reaction to delay, or rigid habit-based responding. Both profiles may be interpreted as reduced or entirely impaired ability to develop a new pattern of choice and/or to modify a previously acquired one. Thus, as an enduring consequence of DAT auto-immune reactions, functional impairment can be proposed for DAT-i mice within the striatum (possibly, its dorso-medial region). In other words, a difficulty in modifying choice strategy and/or a rigid preference profile, shown by DAT-i sub-populations of mice, reflect different degrees of inflexibility due to impaired striatal function. Neuro-chemical data are in agreement with this notion since both subgroups of DAT-i mice showed enhanced HVA (alone or together with DOPAC) in the striatum. Since DA levels were unchanged, it can be proposed that excessive DA metabolism occurred as an enduring adaptation to the auto-immune insult. None of the multiple possibilities can be dismissed (excessive DA synthesis, release or leakage; reduced re-uptake by diminished DAT function or levels); however, an enhanced DA catabolism (with a dynamic limit in HVA fate and its consequent accumulation) seems to be more likely. In some cases, a very big HVA accumulation was denoted by a rise of DOPAC, its intermediate precursor. DOPAC was increased in inflexible (with a “stuck” profile) but not in flexible (with a “slow” profile) DAT-i mice, suggesting that rate of DA metabolism was of an extreme extent in the former subgroup, causing the accumulation of catabolic products deriving from DA.

Moreover, independently from immunization, enhanced products of DA metabolism were found in the PFC of some individuals: specifically, subgroups with a “flat” or a “slow” profile displayed more attraction to LL together with lower HVA in the PFC. Conversely, enhanced DA catabolism may suggest poorer dopaminergic function [72,73], so that cortical control would be worsened, in the subjects with a “steep” or a “stuck” profile. These data point to the role of PFC as subserving inhibitory control over the striatum [74]. The physiological role of PFC is promoting the development of inhibition over impulsive drives, at least in the less flexible sub-population within VEH controls; a reduced PFC function may thus explain the “impulsive” choice strategy shown by the most flexible subjects within VEH controls. Following immunization, and in the presence of a compromised striatal function, the PFC may allow residual learning abilities as observed within flexible DAT-i mice. A relatively reduced function within the PFC, on the other hand, would contribute to the choice-rigidity observed in inflexible subjects among DAT-i mice.

Since the PFC is known to be devoid of DAT, it cannot have been a direct target of the DAT-directed auto-immunity. Such PFC-related features were likely pre-existing across groups. It is noteworthy that we were presently able to identify two separate subgroups of mice, based on their behavioural strategy in the operant choice task: differing levels of flexibility could be associated with diverse DA dynamics in the PFC, and this in the absence of any difference concerning levels of 5-HT nor of its metabolite.

### Implications: auto-immunity in neuro-psychiatry

To interpret the present results, we propose that a transient rise of DAT aAbs, generated by immunization with DAT fragments, induced sequelae onto the striatal complex (possibly in its specific sub-regions), and a long-lasting interference with the corresponding behaviours. Thus, auto-immune reactions, with an increase of circulating aAbs to specific neural targets, may contribute to the etiology of psychiatric symptoms, at least to a certain extent [43-46]. There are many open issues, however, about such a working hypothesis, namely: 1) how can anti-CNS aAbs develop; and 2) how can these aAbs reach their CNS targets and exert their consequences. To cover the first issue (generation of anti-CNS aAbs), two hypotheses can be proposed. First, antibodies generated by immune reactions against an infectious agent may turn out to recognize some epitopes on healthy cells (see [75]), a typical mechanism proposed to account for some neuro-behavioural alterations observed within the so-called pediatric autoimmune neuropsychiatric disorders associated with streptococcal infections (i.e., PANDAS). Second, neuro-receptor fragments may themselves cause generation of auto-immune antibodies, at least in some cases. Indeed, since receptors and proteins expressed by neurons are fragmented during degradation, these fragments may occasionally enter into the bloodstream and act as antigenic peptides [76,77]. This second hypothesis obviously postulates a temporary leakage of blood–brain barrier (BBB).

Such hypothesis should also be involved under the second open issue (i.e., reaching of CNS targets by aAbs), and therefore deserves more investigation. The BBB prevents developing B-cells in the bloodstream from being exposed to unique brain antigens: therefore, there are no mechanisms to establish tolerance to brain antigens and/or to prevent the production of antibodies against them. BBB failure would facilitate drainage of CNS antigens to peripheral lymphoid organs: subsequently, B-cell recognition of CNS antigens would cause: 1) B-cell proliferation and aAbs production; 2) presentation of CNS antigens to T-cells, with interactions between T- and B- cells amplifying the auto-immune response; 3) suggestion that activated B-cells may release aAbs into the circulating bloodstream [28,29]. A related question, about the present anti-DAT aAbs, is then how can they reach the DA pathway, and where the auto-immune insult actually takes place.

As a matter of fact, DAT is not only present in striatal synapses: DA neurons in ventral mesencephalon, i.e. in the Substantia Nigra (SN) pars compacta (SNc; cell group A9) and in the Ventral Tegmental Area (VTA; cell group A10), are enriched with moderate to high DAT immuno-reactive intensity [78,79], where it is concentrated in perikarya, dendrites, and axons. The presence of DAT in the somato-dendritic and axonal compartments of the VTA and the SNc is particularly intriguing, since the nigro-striatal dopaminergic system is specially sensitive to changes in BBB integrity [80], a feature recently associated to Parkinson’s disease (PD). The specific vulnerability of nigral dopaminergic neurons has already been demonstrated in many degeneration models: the Vascular Endothelial Growth Factor (VEGF), Histamine, and Lipo-poly-saccharide (LPS), are all able to disrupt the BBB in the entire ventral mesencephalon, and to reduce cell bodies and fibres of DA neurons in the SNc [81-83]. Previous studies have suggested that microglial density is high in the SNc [84], mediating excessive inflammatory reactions and causing dopaminergic neurons in the SNc to be vulnerable: any transient BBB disruption in the SNc likely results in the vigorous infiltration of neutrophils, which in turn could trigger a vicious cycle of astrocyte and endothelial cell damage, BBB permeability, and further neutrophil infiltration [85]. Therefore, since BBB integrity is crucial to preserve dopaminergic neurons, we propose that a transiently disrupted BBB, in association with immunization procedures, opened the ventral mesencephalon to the action of anti-DAT aAbs, with the permanent consequence of a reduced number of DA cell bodies in the SNc and of subsequent alterations within mesencephalic fibres projecting to the dorsal striatum.

### Limitations and future perspectives

The present results do raise a number of questions which remain unfortunately unanswered. For instance, details about production of DAT aAbs as well as *in vivo* effects of these aAbs on DAT activity are unknown yet (though, check with ref. [86]). Future investigation is warranted to gather full details of antibody development, to verify BBB permeability of such aAbs, and to assess selectivity of effects on DAT activity. We underline that several experimental approaches could be used.

In order to address the first point (i.e., how can DAT aAbs develop in pathogenesis), two main questions should be answered: Are neuro-receptor fragments produced *in vivo* as a side consequence of protein degradation? Are neuro-receptor fragments able to efflux across the BBB? In addition, once postulated that anti-DAT aAbs could be somehow produced, it would be interesting to investigate the BBB influx in the mouse, using isolated/purified and I-125/123/131-radiolabelled anti-DAT aAbs. An alternative way to explore the BBB permeability issue could be to detect these aAbs directly within the brain tissues or at least in the CSF.

Finally, it would be helpful to differentiate between a direct effect (linked to putative DAT protein / anti-DAT aAb interaction) and indirect effects brought by circulating aAbs, as it cannot be excluded that central consequences may arise without the need for these aAbs to cross the BBB. In this line, a direct intra-cerebral injection of these anti-DAT aAbs would allow to study the putative direct effects on functional DAT activity and behaviour. *In vivo* micro-dialysis and fast-scan cyclic voltammetry (FSCV) could be used to assess the actual DA dynamics in brain areas like e.g. the dorsal and ventral striata.

### Concluding remarks

Present findings indicate a profile of slight spontaneous hyperactivity in DAT-i mice, in the form of a 2 h-longer wake-up peak and a wake-up attempt during rest (spontaneous rhythm in their home cages) together with reduced or overtly impaired patterns of flexibility (operant choice task in Skinner-box cages). It is well known that the ventral portion of the striatum subserves a motivational drive to flexibly change choice strategies [58,60,64]. After ventral striatum has provided motivation, such behavioural flexibility requires the recruitment of specific circuits within dorsal striatum [59,62]; if these are compromised, the expression of habits is observed, whereby semi-automatic behaviour does not respond to devaluation of its outcomes [61,63].

Our data are consistent with a functional impairment of the striatal complex, as a long-lasting consequence tapping onto the dopaminergic pathways and owing to the DAT immunization. Namely, the transient (but detectable) rise in circulating aAbs, generated by specific neuro-receptor fragments used as antigen, possibly together with a concomitantly permeable BBB (occasionally possible in stressful conditions; see [87]), might have generated persistent neuro-behavioural sequelae. Similarly, in a recent study [49], mice specifically immunized with fragments of glutamate receptors (GluR1) also demonstrated altered spontaneous activity and a stereotyped behavioural syndrome in response to a novelty-induced stress. A possible limitation in our study may be the lack of a further control group, immunized with a non-specific peptide. It is not easy however to figure out what this control peptide could be, since the exact sequence of the DAT antigens is currently protected information.

In conclusion, brain-directed auto-immune responses are likely to interfere with (or produce enduring impairments to) function of brain areas, with all the consequences thereof. More research effort is needed to verify potential psycho-immune alterations, via changes in the brain and behaviour of immunized mice. An altered psycho-immune modulation of neuro-receptors, like GluR1 or DAT, may play a role in vulnerability to neuro-psychiatric disorders like ADHD and OCD, as well as other impulse-control disorders like e.g. pathological gambling and sensation seeking.

## Abbreviations

“flat” profile: inflexible subgroup within VEH mice; “steep” profile: flexible subgroup within VEH mice; “slow” profile: flexible subgroup within DAT-i mice; “stuck” profile: inflexible subgroup within DAT-i mice.

## Competing interests

There is no conflict of interest to disclose.

## Authors’ contribution

WA and GL planned the experiment and interpreted data; SK and SCC carried out the immunization and behavioural experiments; ER and DT realized the ELISAs for DAT aAbs; RvdB gave advice about behavioural data and discussion; OG provided the DAT antigens for immunization; SFA realized the neurochemical analyses ex-vivo. All authors read and approved the final manuscript.
